# Role of Efflux Pump-Mediated Antibiotic Resistance in Quorum Sensing-Regulated Biofilm Formation by *Salmonella* Typhimurium

**DOI:** 10.3390/pathogens11020147

**Published:** 2022-01-24

**Authors:** Jirapat Dawan, Yinyue Li, Feng Lu, Xinlong He, Juhee Ahn

**Affiliations:** 1 Department of Biomedical Science, Institute of Bioscience and Biotechnology, Kangwon National University, Chuncheon 24341, Gangwon, Korea; jirapat@kangwon.ac.kr; 2 Institute of Translational Medicine, Medical College, Yangzhou University, Yangzhou 225001, China; lyy1242602847@163.com (Y.L.); lufeng@yzu.edu.cn (F.L.); hexl@yzu.edu.cn (X.H.)

**Keywords:** *Salmonella*, biofilm, antibiotic susceptibility, efflux pump inhibitor

## Abstract

This study was designed to assess the influence of efflux pump activity on the biofilm formation in *Salmonella* Typhimurium. *Salmonella enterica* subsp. *enterica* serovar Typhimurium ATCC 19585 (ST^WT^) and clinically isolated *S.* Typhimurium CCARM 8009 (ST^CI^) were treated with ceftriaxone (CEF), chloramphenicol (CHL), ciprofloxacin (CIP), erythromycin (ERY), norfloxacin (NOR), and tetracycline (TET) in autoinducer-containing media in the absence and presence of phenylalanine-arginine β-naphthylamide (PAβN) to compare efflux pump activity with biofilm-forming ability. The susceptibilities of ST^WT^ and ST^CI^ were increased in the presence of PAβN. ERY+PAβN showed the highest decrease in the minimum inhibitory concentration (MIC) of ERY from 256 to 2 μg/mL against ST^WT^ and ST^CI^. The antimicrobial activity of NOR against planktonic cells was significantly increased in the presence of PAβN, showing the lowest numbers of ST^WT^ (3.2 log CFU/cm^2^), and the TET+PAβN effectively inhibited the growth of ST^CI^ (5.2 log CFU/cm^2^). The lowest biofilm-forming abilities were observed at NOR+PAβN against ST^WT^ (biofilm-forming index, BFI < 0.41) and CEF+PAβN against ST^CI^ (BFI = 0.32). The bacteria swimming motility and relative fitness varied depending on the antibiotic and PAβN treatments. The motility diameters of ST^WT^ were significantly decreased by NOR+PAβN (6 mm) and TET+PAβN (15 mm), while the lowest motility of ST^CI^ was observed at CIP+PAβN (8 mm). The significant decrease in the relative fitness levels of ST^WT^ and ST^CI^ was observed at CIP+PAβN and NOR+PAβN. The PAβN as an efflux pump inhibitor (EPI) can improve the antimicrobial and anti-biofilm efficacy of antibiotics against *S*. Typhimurium. This study provides useful information for understanding the role of efflux pump activity in quorum sensing-regulated biofilm formation and also emphasizes the necessity of the discovery of novel EPIs for controlling biofilm formation by antibiotic-resistant pathogens.

## 1. Introduction

Over the last few decades, antibiotics have been indiscriminately overused and misused in the treatment of infectious diseases. However, the extensive use of antibiotics has accelerated the emergence and spread of multidrug-resistant pathogens, leading to frequent treatment failure due to the limited chemotherapeutic options [1]. Salmonella Typhimurium is highly resistant to various antibiotic classes such as β-lactams and fluoroquinolones, resulting in treatment failure and increased morbidity and mortality [2]. The acquired multidrug resistance in bacteria can be caused by many mechanisms, including reduced membrane permeability, enzymatic modification, target site alteration, protective shield formation, and efflux pump activity [3]. Among these mechanisms, the efflux pumps are known primarily to confer multidrug resistance in bacteria [4]. The multidrug-resistant bacteria can expel different classes of antibiotics through well-developed efflux pump systems such as adenosine triphosphate (ATP)-binding cassette (ABC) superfamily, major facilitator superfamily (MFS), small multidrug resistance (SMR) family, multidrug and toxic compound extrusion (MATE) family, and resistance nodulation division (RND) superfamily [4,5]. The RND efflux pumps as polyspecific transporters are directly responsible for multidrug resistance in *Salmonella* strains [6].

The antibiotic substrates of single-component efflux pumps pass through the inner membrane into the periplasmic space in Gram-negative bacteria, and the transported antibiotic substrates traverse the outer cell membrane through multiple-component efflux pumps consisting of an inner membrane transporter, periplasmic membrane fusion, and β-barrel channel proteins [7]. In addition, the multidrug efflux pump system has drawn increasing attention as a potential therapeutic target to control biofilm formation [3]. A bacterial cell-to-cell communication system, regulated by a cell density-dependent manner, known as quorum sensing (QS), coordinates the formation of biofilm cells that are highly resistant to antibiotics [3]. *Chromobacterium violaceum* is commonly known as an acyl-homoserine lactone (AHL) producer, which regulates the production of proteases or pigment compounds such as violacein [8]. The AHLs secreted from *C*. *violaceum* can transfer across bacterial cells and induce the QS-controlled virulence and pathogenicity [8]. In addition, the multidrug efflux pumps are involved in the secretion of QS-signaling molecules such as autoinducer 1 (AI-1) and autoinducer 2 (AI-2), which are involved in biofilm formation [5]. Furthermore, the efflux pump-related genes are more expressed in biofilm cells than planktonic cells [9,10]. The overexpression of efflux-related genes corresponds to the overexpression of QS-related genes [11]. Efflux pump inhibitors (EPIs) can block the transport of QS-signaling molecules across membrane channels, resulting in the disruption of biofilm formation [3,12]. Hence, the inhibition of efflux pump expression plays an important role in controlling biofilm formation regulated by QS [13]. However, there is still a lack of knowledge regarding the effect of multidrug efflux pumps on the biofilm formation. Therefore, the aims of this study were to evaluate the antibiotic susceptibility and biofilm-forming ability of *Salmonella* Typhimurium ATCC 19585 (ST^WT^) and clinically isolated antibiotic-resistant *S.* Typhimurium CCARM 8009 (ST^CI^) in the presence of phenylalanine-arginine β-naphthylamide (PAβN) and also investigate the relationship between efflux pump activity and biofilm formation of ST^WT^ and ST^CI^ in *C*. *violaceum*-cultured cell-free supernatant (CFS).

## 2. Results

### 2.1. Antibiotic Susceptibilities of Salmonella Typhimurium Exposed to Efflux Pump Inhibitor

**Figure 1:**
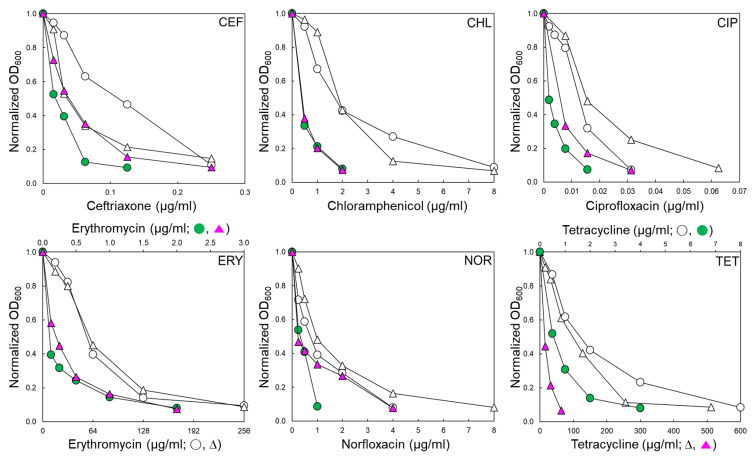
Antibiotic susceptibility of *Salmonella* Typhimurium ATCC 19585 (ST^WT^; ○, ●) and clinically isolated antibiotic-resistant *S.* Typhimurium CCARM 8009 (ST^CI^; ∆, ▲) treated with ceftriaxone (CEF), chloramphenicol (CHL), ciprofloxacin (CIP), erythromycin (ERY), norfloxacin (NOR), and tetracycline (TET) in the absence (○, ∆) and presence (●, ▲) of phenylalanine-arginine β-naphthylamide (PAβN).

ST^WT^ and ST^CI^ used in this study have different antibiotic resistance profiles, as shown in Table 1. The antibiotic susceptibilities of ST^WT^ and ST^CI^ were evaluated in the absence and presence of EPI, phenylalanine-arginine β-naphthylamide (PAβN 120 μg/mL) (Table 1). The antibiotic susceptibility patterns of ST^WT^ and ST^CI^ exposed to PAβN varied in the classes of antibiotics. The antibiotic activities of ceftriaxone (CEF), chloramphenicol (CHL), ciprofloxacin (CIP), erythromycin (ERY), norfloxacin (NOR), and tetracycline (TET) against ST^WT^ and ST^CI^ were increased in the presence of PAβN (Figure 1). The minimum inhibitory concentration (MIC) of CEF against ST^WT^ was decreased from 0.25 to 0.125 μg/mL when treated with PAβN. Although the growth of ST^CI^ was slightly decreased at CEF+PAβN, the addition of PAβN did not affect the MIC of CEF against ST^CI^. The MICs of CHL against ST^WT^ and ST^CI^ were decreased from 8 to 2 μg/mL in the presence of PAβN. The susceptibilities of ST^WT^ and ST^CI^ to CIP, ERY, NOR, and TET were increased in the presence of PAβN. The ERY+PAβN showed the highest decrease in the MIC from 256 to 2 μg/mL toward ST^WT^ and ST^CI^. The highest MIC was observed for ST^CI^ treated with TET (512 μg/mL) and was significantly decreased in the presence of PAβN (Figure 1).

### 2.2. Biofilm-Forming Abilities of Salmonella Typhimurium Treated with Antibiotics in the Presence of PAβN

**Figure 2:**
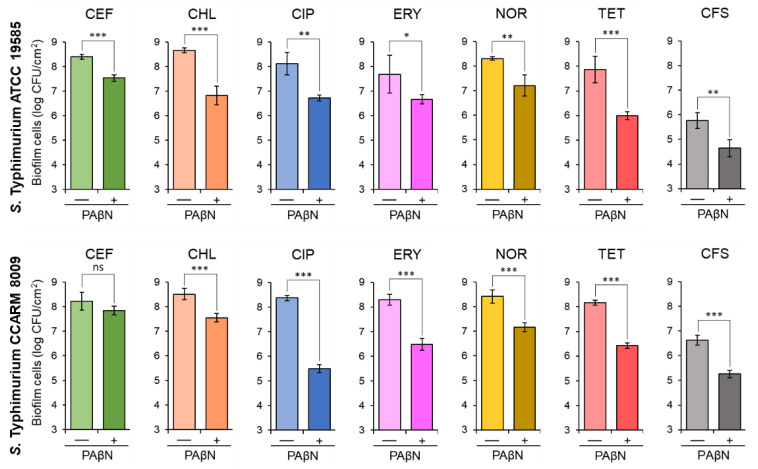
Biofilm-forming abilities of *Salmonella* Typhimurium ATCC 19585 (ST^WT^) and clinically isolated antibiotic-resistant *S.* Typhimurium CCARM 8009 (ST^CI^) treated with 1/2 MICs of ceftriaxone (CEF), chloramphenicol (CHL), ciprofloxacin (CIP), erythromycin (ERY), norfloxacin (NOR), and tetracycline (TET) in the absence (−) and presence (+) of phenylalanine-arginine β-naphthylamide (PAβN) and the biofilm-forming abilities of ST^WT^ and ST^CI^ in *Chromobacterium violaceum*-cultured cell-free supernatant (CFS) in the absence (−) and presence (+) of PAβN. ns, *, **, and *** indicate no significant difference (*p* > 0.05) and the significant difference between absence and presence of PAβN at *p* < 0.05, *p* < 0.01, and *p* < 0.001, respectively.

The biofilm-forming abilities of ST^WT^ and ST^CI^ were evaluated at the sub-MICs of antibiotics, CEF (0.02 and 0.03 μg/mL), CHL (0.5 μg/mL), CIP (0.004 and 0.008 μg/mL), ERY (0.5 μg/mL), NOR (0.25 and 1 μg/mL), and TET (1 and 8 μg/mL), respectively, in the absence and presence of PAβN. The sub-MICs of CEF, CHL, CIP, ERY, NOR, and TET were not effective on the inhibition of biofilm formation of ST^WT^ and ST^CI^, which were considerably inhibited in the presence of PAβN (Figure 2). The numbers of biofilm cells formed by ST^WT^ and ST^CI^ treated with antibiotics alone were approximately 8 log CFU/cm^2^. However, the addition of PAβN significantly decreased the biofilm-forming abilities of ST^WT^ and ST^CI^, showing the noticeable reductions in biofilm cell numbers when treated with CEF+PAβN, CHL+PAβN, CIP+PAβN, ERY+PAβN, NOR+PAβN, and TET+PAβN (Figure 2). In addition, the biofilm-forming abilities of ST^WT^ and ST^CI^ were evaluated in the cell-free supernatant (CFS) of *Chromobacterium violaceum*-cultured media with or without PAβN. The number of ST^WT^ and ST^CI^ biofilm cells in CFS without PAβN were 5.8 and 6.6 log CFU/cm^2^, respectively, after 24 h of incubation at 37 °C, while the numbers of ST^WT^ and ST^CI^ biofilm cells in CFS were significantly decreased when treated with PAβN (Figure 2).

### 2.3. Viability of Salmonella Typhimurium Treated with Antibiotic and PAβN

**Figure 3:**
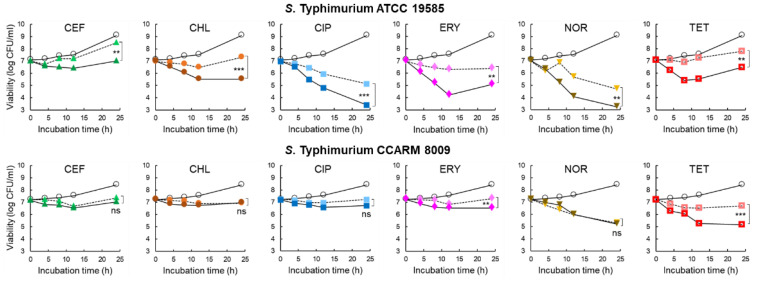
Survival of *Salmonella* Typhimurium ATCC 19585 (ST^WT^) and clinically isolated antibiotic-resistant *S.* Typhimurium CCARM 8009 (ST^CI^) treated with 1/2 MICs of ceftriaxone (CEF), chloramphenicol (CHL), ciprofloxacin (CIP), erythromycin (ERY), norfloxacin (NOR), and tetracycline (TET) in *Chromobacterium violaceum*-cultured cell-free supernatant (CFS) [○; untreated control, without (▲, ●, ■, ♦, ▼, 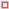), and with (▲, ●, ■, ♦, ▼, 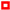) phenylalanine-arginine β-naphthylamide (PAβN)]. ns, **, and *** indicate no significant difference (*p* > 0.05) and the significant difference between absence and presence of PAβN at *p* < 0.05, *p* < 0.01, and *p* < 0.001, respectively.

The inhibitory effect of 1/2 MICs of CEF (0.125 and 0.25 μg/mL), CHL (2 and 8 μg/mL), CIP (0.015 and 0.031 μg/mL), ERY (64 and 128 μg/mL), NOR (1 and 4 μg/mL), and TET (4 and 256 μg/mL) on the growth of ST^WT^ and ST^CI^, respectively, was evaluated in the CFS of *C. violaceum*-cultured media with or without PAβN for 24 h at 37 °C. As shown in Figure 3, all antibiotic treatments significantly inhibited the growth of ST^WT^ at the early stage of incubation (<12 h). The numbers of ST^WT^ treated with CEF and CHL were increased at the late stage of incubation, showing 8.5 and 7.3 log CFU/cm^2^ after 24 h of incubation, respectively. However, the antibiotic activities of CEF and CHL against ST^WT^ were enhanced by PAβN, showing 7.0 log CFU/cm^2^ at CEF+PAβN and 5.5 log CFU/cm^2^ at CHL+PAβN, respectively. Compared to the control, CIP+PAβN most effectively inhibited the growth of ST^WT^ (3.4 log CFU/cm^2^) after 24 h of incubation. PAβN restored the antimicrobial activity of ERY against ST^WT^, showing 4.2 log CFU/cm^2^ at 12 h of incubation. However, the bacterial re-growth was observed for ERY and ERY+PAβN at 24 h of incubation. ST^WT^ was susceptible to NOR in the presence of PAβN, showing the lowest number of 3.2 log CFU/cm^2^. The growth of ST^WT^ was significantly decreased by TET (6.9 log CFU/cm^2^) and TET+PAβN (5.4 log CFU/cm^2^) at 8 h of incubation. However, the rapid growth of ST^WT^ was observed at TET and TET+ PAβN after 12 h of incubation, showing 7.8 and 6.5 log CFU/cm^2^, respectively (Figure 3).

No significant difference in the antimicrobial activities was observed for CEF (7.3 log CFU/cm^2^) and CEF+PAβN (7.0 log CFU/cm^2^) against ST^CI^ after 24 h of incubation. The numbers of ST^CI^ had no significant difference between CHL and CHL+PAβN at the late stage of incubation, showing 7.0 log CFU/cm^2^ at 24 h of incubation. Compared to the control, the significant decrease in the numbers of ST^CI^ was observed at CIP and CIP+PAβN treatments, showing no significant difference between CIP (7.2 log CFU/cm^2^) and CIP+PAβN (6.7 log CFU/cm^2^) at 24 h of incubation. PAβN enhanced ERY activity and inhibited the growth of ST^CI^ up to 24 h of incubation, showing 6.5 log CFU/cm^2^ at ERY+PAβN. Compared to the control, the growth of ST^CI^ was rapidly decreased to 5.3 and 5.2 log CFU/cm^2^ by NOR and NOR+PAβN, respectively, after 24 h of incubation. The growth of ST^CI^ was significantly decreased by TET+PAβN up to 12 h (5.2 log CFU/cm^2^), which is the most effective treatment to inhibit the growth of ST^CI^, whereas the steady growth was observed after 12 h of incubation, showing 5.1 log CFU/cm^2^ (Figure 3).

### 2.4. Biofilm-Forming Ability, Motility, and Relative Fitness of Salmonella Typhimurium Cultured in Autoinducer-Containing Media

**Figure 4:**
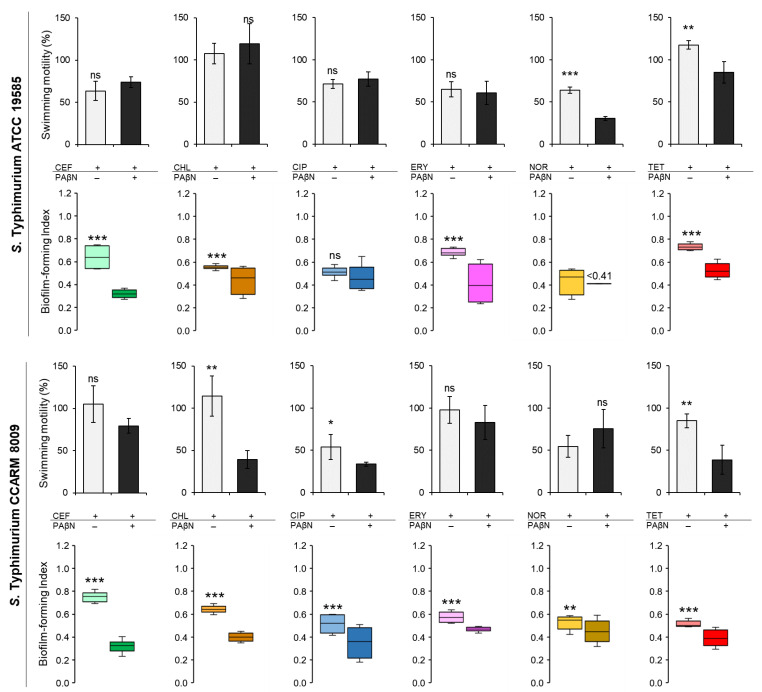
The biofilm-forming index (BFI) and swimming motility of *Salmonella* Typhimurium ATCC 19585 (ST^WT^) and clinically isolated antibiotic-resistant *S.* Typhimurium CCARM 8009 (ST^CI^) treated with 1/2 MICs of ceftriaxone (CEF), chloramphenicol (CHL), ciprofloxacin (CIP), erythromycin (ERY), norfloxacin (NOR), and tetracycline (TET) in *Chromobacterium violaceum*- cultured cell-free supernatant (CFS). (BFI in the absence (■, ■, ■, ■, ■, ■) and presence (■, ■, ■, ■, ■, ■) of phenylalanine-arginine β-naphthylamide (PAβN) and swimming motility in the absence (□) and presence (■) of PaβN). ns, *, **, and *** indicate no significant difference (*p* > 0.05) and the significant difference between the absence and presence of PAβN at *p* < 0.05, *p* < 0.01, and *p* < 0.001, respectively.

The biofilm-forming abilities and the swimming motilities of ST^WT^ and ST^CI^ were evaluated in CFS when treated with CEF, CHL, CIP, FRY, NOR, and TET in the absence and presence of PAβN (Figure 4). The biofilm-forming index (BFI < 0.64) of ST^WT^ at the combination treatments of antibiotics and PAβN was noticeably decreased when compared to antibiotic alone in the exception of CIP (Figure 4). The presence of PAβN can enhance the anti-biofilm activity of CEF, CHL, CIP, ERY, NOR, and TET against ST^WT^, showing the decrease in BFI to 0.32 at CEF+PAβN, 0.44 at CHL+PAβN, 0.47 at CIP+PAβN, 0.41 at ERY+PAβN, less than 0.41 at NOR+PAβN, and 0.53 at TET+PAβN, respectively. The motility of ST^WT^ was varied in treatments. The swimming motilities of ST^WT^ treated with antibiotics alone, including CEF (11 mm), CHL (19 mm), CIP (13 mm), and ERY (12 mm), were not significantly different from CEF+PAβN (13 mm), CHL+PAβN (22 mm), CIP+PAβN (14 mm), and ERY+PAβN (11 mm), respectively. However, the motility diameters of ST^WT^ were significantly decreased by NOR+PAβN (6 mm) and TET+PAβN (15 mm) when compared to NOR (12 mm) and TET (21 mm) (Figure 4).

Compared to the control, the BFI of ST^CI^ was decreased in the combination treatments of antibiotics and PAβN (Figure 4). The BFI values of ST^CI^ treated with CEF, CHL, CIP, ERY, NOR, and TET were 0.75, 0.64, 0.51, 0.57, 0.53, and 0.51, respectively, while those were decreased to 0.32, 0.40, 0.35, 0.47, 0.45, and 0.39, respectively, after the combination treatments. Regardless of the absence and presence of PAβN, no significant differences in the swimming motility of ST^CI^ were observed for each treatment, showing at CEF (24 mm), CEF+PAβN (18 mm), ERY (22 mm), ERY+PAβN (19 mm), NOR (12 mm), and NOR+PAβN (17 mm). The significant decrease in swimming motility of ST^CI^ was observed at CHL (26 mm), CHL+PAβN (9 mm), CIP (12 mm), CIP+PAβN (8 mm), TET (19 mm), and TET+PAβN (9 mm) (Figure 4). The relative fitness levels of ST^WT^ and ST^CI^ treated with CEF, CHL, ERY, and TET were more than 0.9 and 0.7, respectively, which were decreased when treated with PAβN (data not shown). The noticeable reduction in relative fitness of ST^WT^ and ST^CI^ was observed at CIP+PAβN and NOR+PAβN.

### 2.5. Correlation between Planktonic Growth and Biofilm Formation of Salmonella Typhimurium Treated with Antibiotics and PAβN

**Figure 5:**
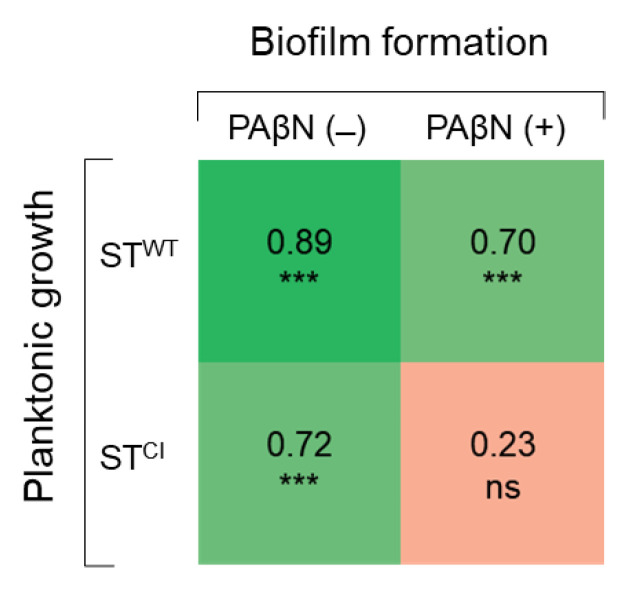
Correlation matrix of Pearson coefficients between planktonic cell count and biofilm cell counts (*n* = 24) of *Salmonella* Typhimurium ATCC 19585 (ST^WT^) and clinically isolated antibiotic-resistant *S.* Typhimurium CCARM 8009 (ST^CI^) in the absence and presence of PAβN. ns and *** indicate no significance (*p* > 0.05), significant difference at *p* < 0.05, *p* < 0.01, and *p* < 0.001, respectively.

The correlation coefficients between the numbers of planktonic and biofilm cells of ST^WT^ and ST^CI^ were compared in the absence and presence of PAβN (Figure 5). The planktonic cell numbers were positively correlated with the biofilm cell numbers of ST^WT^ (*r* = 0.89) and ST^CI^ (*r* = 0.72) in the absence of PAβN. ST^WT^ planktonic and biofilm cells in the presence of PAβN were highly correlated at 0.70. However, no correlation between ST^CI^ planktonic cells and biofilm cells was observed in the presence of PAβN (*r* = 0.23).

## 3. Discussion

This study describes the interplay between efflux pump activity and biofilm formation of ST^WT^ and ST^CI^. The bacterial efflux pumps are transmembrane transport proteins targeting multiple substrates, which are known as major determinants of multidrug resistance in bacteria [14]. The RND family is a major efflux pump system in Gram-negative bacteria, which is responsible for intrinsic and acquired resistance to various antibiotics. The AcrAB-TolC belonging to the RND efflux system is the most abundant efflux pump in *S*. Typhimurium [6]. The loss of AcrB in *S.* Typhimurium leads to the enhanced susceptibility to quinolones, tetracycline, and chloramphenicol, and the overexpression of *acrB* contributes to the reduced susceptibility to those antibiotics [15]. In general, AcrB is a substrate-binding trimer anchored in the inner membrane that extends into the periplasm and links with the outer membrane protein TolC, while AcrA is an accessory protein of the functional system [16]. AcrAB-TolC has a binding affinity to multi-substrates such as hydrophilic antibiotics and negatively charged β-lactams [17]. Therefore, the efflux pump systems contribute to the development of antibiotic resistance and also play an important role in the formation of bacterial biofilms [5,13,18]. Several studies have been reported that EPI could be used in a control strategy to inhibit biofilm formation [18,19]. The inhibition of efflux pumps can reduce biofilm formation by interfering with QS-mediated communication within and between strains [20,21,22]. PAβN, a broad-spectrum peptidomimetic inhibitor, inhibits RND efflux pump systems such as AcrAB-TolC of *S*. Typhimurium [13]. The PAβN competitively binds to the hydrophobic trap of AcrA and AcrB to interrupt the substrate transport pathway [13]. Furthermore, membrane integrity and lipopolysaccharide (LPS) are major target-sites for PAβN, specifically RND-type AcrAB–TolC and MexAB efflux pumps [14]. Therefore, the EPIs can potentiate antibiotic activity against antibiotic-resistant Gram-negative bacteria [23].

The MICs of CEF, CHL, CIP, ERY, NOR, and TET against ST^WT^ and ST^CI^ were considerably decreased in the presence of PAβN (Figure 1). These results confirm that the efflux pumps are involved in the intrinsic resistance of *S*. Typhimurium to several classes of antibiotics. This suggests that the antibiotic resistance in *S*. Typhimurium is associated with the substrate competition-dependent efflux systems [24]. The PAβN noticeably enhanced the ERY activities against ST^WT^ and ST^CI^, suggesting that an intrinsic efflux pump activity might use ERY as substrate, leading to ERY resistance. This observation is in good agreement with a previous report that PAβN exhibited the enhanced ERY susceptibility against Gram-negative bacteria [25]. The increased susceptibility to ERY may be attributed to the inhibition of RND efflux, MacAB-TolC, in the presence of PAβN, which is the main resistance mechanism of ERY in *S*. Typhimurium [26]. The antimicrobial activities of CIP, NOR, and TET against ST^WT^ and ST^CI^ were enhanced in the presence of PAβN. This observation is in good agreement with a previous report that the MICs of quinolones and tetracyclines were decreased when treated with EPI [25]. Moreover, the deletion of AcrAB-TolC genes resulted in four-fold reduction in the MIC of chloramphenicol, suggesting that AcrAB and TolC proteins, which belong to RND efflux, are important for the development of antibiotic resistance of *S*. Typhimurium [27]. The overexpression of *mdfA* and *norE* could contribute to fluoroquinolone resistance under the overproduction of RND and AcrAB-TolC. In general, the overproduction of AcrAB-TolC increases the MICs of several antibiotics against bacteria. In addition, tetracycline is transported by the MFS and RND efflux systems [28]. This suggests that the inhibition of the RND efflux pump system would be the major contribution to acquiring quinolones and tetracyclines resistance in *S*. Typhimurium. The major requirement for substrates of the RND efflux pump system is an insertion into the membrane bilayer. For instance, lipophilic side chains are likely to be partitioned into the lipid bilayer of the cytoplasmic membrane, being substrates of the RND efflux pump system [29]. Therefore, the EPIs could effectively enhance the susceptibility to lipophilic antibiotics including chloramphenicol, quinolone, macrolide, and tetracycline. Moreover, the efflux pumps belonging to the RND family are known, as the most effective efflux pump system extrude toxic compounds in *S.* Typhimurium [15]. Therefore, the competitive binding of PAβN to the RND efflux pump can help control the multidrug-resistant bacteria.

Bacterial biofilms are highly resistant to environmental and chemical stresses such as heat, desiccation, acid, osmotic stress, food preservatives, disinfectants, and antibiotics [4]. The structural integrity of the complex biofilm matrix consisting of DNA, polysaccharides, and proteins provides membrane permeability barrier to antibiotics [30]. Indeed, biofilms are able to induce antibiotic resistance through several mechanisms including permeability disruption, starvation–stress response, and efflux pump activation [31]. In addition, the active efflux and diffusion can be involved in the transport of QS-signaling molecules, leading to the development of biofilm formation in Gram-negative bacteria [3]. The endogenous substrates of RND efflux pumps include fatty acids, natural antibiotics, QS-signaling molecules, and QS precursors [6,32,33]. In general, the biofilm formation of *S*. Typhimurium is regulated by cell communication process, which is called QS [34]. In a high cell density, bacteria produce and recognize QS-signaling acyl-homoserine lactones (AHLs), called autoinducer (AI), to coordinate gene expression [34]. The extrageneous AI-1 synthesized by various strains and *Salmonella* self-producing AI-2 can facilitate intra- and interspecies communication within *S*. Typhimurium biofilms. Therefore, quorum quenching (QQ) can be a possible approach to control bacterial biofilm formation in that QS inhibitors interrupt bacterial communication. Recently, the QQ has drawn more attention to enhance antibiotic susceptibility and eventually inhibit biofilm formation. The biofilm-forming abilities of ST^WT^ and ST^CI^ were inhibited by CEF, CHL, CIP, ERY, NOR, and TET in the presence of PAβN (Figure 2). The inhibition of biofilm-forming ability resulted in increased antibiotic activity because of the inactivation of efflux pumps developed in ST^WT^ and ST^CI^. The sub-MICs of CEF, CHL, CIP, ERY, NOR, and TET can increase the protein level in bacterial membrane compositions and exopolysaccharide production, leading to the antibiotic-induced biofilm formation [35]. However, the addition of PAβN restored the antibiotic activity against ST^WT^ and ST^CI^ (Figure 2). This suggests that the efflux pumps can use QS-signaling molecules as substrates and eventually inhibit the biofilm formation of *S*. Typhimurium. The deletion of efflux pump-related genes such as *acrB* and *tolC* could cause the loss of curli and extracellular matrix production [36]. The CEF activity against ST^WT^ and ST^CI^ was increased in the presence of PAβN (Figure 2). This is in good agreement of previous report that ABC transporter is involved in the resistance to β-lactam antibiotics, corresponding to the role of AcrAB–TolC in the enhanced resistance to β-lactam antibiotics [37]. In addition, the RND-type efflux pump, MdsABC, has a broad substrate range, including chloride, cephalosporin, chloramphenicol, and novobiocin [38]. Therefore, the RND efflux pump plays a major role in biofilm formation [16]. The efflux pump-assisted biofilm formation is attributed to the expulsion of extracellular polymeric substances (EPSs) and QS signaling molecules, the regulation of biofilm-related genes, efflux of toxic components, and prevention of cell-to-cell and cell-to-surface adhesion [13,39]. This implies that the RND efflux pump inhibited by PAβN could retard the biofilm formation. The CIP+PAβN and NOR+PAβN decreased the biofilm-forming abilities of ST^WT^ and ST^CI^ (Figure 2). The QeqA and OqxAB sharing homology with AcrAB and MexAB efflux systems have the ability to hydrolyze fluoroquinolones [40]. Therefore, the reduced biofilm numbers in ST^WT^ and ST^CI^ treated with CIP or NOR and PAβN might be attributed to QeqA and OqxAB that are specific for fluoroquinolone. This suggests that PAβN could reduce quinolone resistance and restore the quinolone antibiotic activity against Gram-negative bacteria [41]. Moreover, the addition of PAβN also increased the anti-biofilm activity of CHL, ERY, and TET. The reduced biofilm numbers of ST^WT^ and ST^CI^ treated with CHL, ERY, and TET in the presence of PAβN might be due to the inhibition of mexAB-oprM. This observation implied that the mexAB-oprM efflux pump system is linked to intrinsic resistance to antibiotics such as chloramphenicol, quinolones, macrolides, and tetracycline [42]. MexAB-OprM and MexEF-OprN in Gram-negative bacteria can transport intercellular signals or intermediates during biofilm formation [43]. Therefore, the inhibition of efflux pumps might be a good strategy to effectively inhibit biofilm formation.

Gram-negative bacteria synthesize various *N*-acyl homoserine lactones (AHLs) in a cell density-dependent manner [44]. In this study, *Salmonella* can detect AHLs produced by *C. violaecium* [45]. *Salmonella* spp. cannot synthesize their own AHLs because of the absence of a luxI homolog but can recognize external AHLs by the receptor, SdiA [44]. The results as given in Figure 2 imply that the biofilm numbers were reduced in CFS containing AHLs when treated with PAβN. This observation suggests that PAβN blocked the transport of AHLs into ST^WT^ and ST^CI^, leading to the inhibition of biofilm formation. The RND efflux pump can extrude AHLs such as 3OC_12_-HSL [46]. For instance, MexCD-OprJ can extrude AIs, resulting in the reduced accumulation of AIs in the bacterial cell [46]. Thus, the inhibition of efflux pumps could affect the AHL transport, leading to the reduced expression of AHL-based QS genes and decrease in biofilm formation.

The sub-MICs of CEF, CHL, CIP, ERY, NOR, and TET could not inhibit the growth of ST^WT^ and ST^CI^. No significant differences in the bacterial numbers between the control and antibiotic treatments (Figure 3). The growths of CEF-, CHL-, ERY-, NOR-, and TET-injured ST^WT^ and CEF-, CHL-, CIP-, and ERY-stressed ST^CI^ were recovered after 12 h, but the recovery rates were significantly lowered in the presence of PAβN. The growths of ST^WT^ and ST^CI^ were most effectively inhibited by the antibiotics combined with PAβN throughout the incubation period. The decrease in the numbers of bacteria treated with the combination of antibiotics and EPI suggests that the efflux pumps are associated with the innate resistance in *S.* Typhimurium. However, the mechanisms of resistance of bacteria to β-lactams and chloramphenicol are also associated with β-lactamases-mediated hydrolysis, porin channels, and efflux pump activity [47]. The change in the porin channel influences membrane permeability as an access barrier to antibiotics such as β-lactams and quinolones [47]. The growths of ST^CI^ treated with CEF, CEF+PAβN, CHL, and CHL+PAβN were steady constant regardless of the levels of efflux pump activities (Figure 3). Compared to the control, the numbers of ST^WT^ and ST^CI^ treated with CIP, ERY, and TET were reduced in the presence of PAβN up to 12 h of incubation (Figure 4). This result implies that the efflux pump can extrude antibiotics such as macrolides, tetracyclines, and quinolones as substrates. Moreover, the mechanism of resistance of bacteria to fluoroquinolones is caused by mutations in DNA-gyrase and topoisomerase IV, altered permeability, and active efflux pump [48]. The fluoroquinolone efflux pump system of *S*. Typhimurium is encoded by the AcrAB homologous protein [49]; thus, PAβN can effectively enhance the antibiotic activities of CIP and NOR against ST^WT^ and ST^CI^ (Figure 3).

The biofilm-forming abilities of ST^WT^ and ST^CI^ in CFS containing AHLs are shown in Figure 4. The combination treatments of antibiotics and PAβN showed a lower biofilm-forming index (BFI) than the single antibiotic treatments (Figure 4). The combination of antibiotics and PAβN can synergistically inhibit QS, resulting in the inhibition of biofilm formation. This observation is in good agreement with previous study that the combinations of EPI with fluoroquinolones effectively inhibited QS and biofilm formation [50]. The inhibition of efflux pump in CFS media may be related to the decrease in transport of AHLs, leading to the inhibition of biofilm formation. The lowest BFI was observed in ST^CI^ treated with CEF+PAβN (0.32), which was followed by CIP+PAβN (0.35) and TET+PAβN (0.39) (Figure 4). This indicates that EPIs are effective at inhibiting biofilm formation under low concentrations of antibiotics and even enhance the therapeutic potential of low doses of antibiotics. The extrusion of QS-signaling molecules causes an increased concentration of extracellular self-inducers, leading to serious bacterial infection [51]. Thus, certain efflux pumps have a role in modifying both the QS response and pathogenicity in bacteria [18]. In addition, during the infection, pathogenic bacteria can produce a variety of virulence factors such as toxins, outer membrane proteins, or biofilm to invade the host or evade the host immune system [52]. Among virulence factors produced by Gram-negative bacteria, the production of QS-regulated virulence factors plays an important role in biofilm formation. For example, the production of pyocyanin and rhamnolipids from the QS system is responsible for the deposition of extracellular DNA [19]. This indicates that the high efflux pump activity may promote the QS system, leading to the synthesis of several virulence factors and the increase in bacterial pathogenicity. This confirms that the bacterial efflux pumps and QS system are involved in the regulation of biofilm formation. Therefore, the inhibition of bacterial efflux pumps can restore antibiotic activity, leading to the disruption of biofilm formation [14].

ST^WT^ treated with NOR+PAβN showed the lowest motility when compared to other treatments (Figure 4). At the early stages of biofilm formation, swimming motility plays an important role in bacterial translocation and adhesion to the surface [53]. This indicates that the reduced motility in the presence of PAβN may result in the decreased ability of ST^WT^ to form biofilms. However, the lowest swimming motility of ST^CI^ was observed at CIP+PAβN, but the lowest BFI was observed at CEF+PAβN (Figure 4). This implies that the swimming motility was not directly correlated with the biofilm-forming ability of ST^CI^ in the presence of PAβN. The relative fitness levels of ST^WT^ and ST^CI^ treated with antibiotics were comparably decreased in the presence of PAβN, in other words reflecting the increase in fitness cost. In general, bacteria can quickly adapt to new challenges and subsequently continue to optimize their fitness [54]. The antibiotic-resistant bacteria have relatively low fitness under favorable conditions [55]. In addition, bacteria with low efflux pump activity might have a higher fitness cost than bacteria with high efflux pump activity [56]. In particular, genes involved in peptidoglycan synthesis or efflux control were detected more often in populations with low fitness [57]. Therefore, bacteria with a low fitness cost are more likely to develop antibiotic resistance because of a fitness advantage [54]. Furthermore, the accumulation of compensatory mutations can restore the bacterial fitness and decrease the fitness cost in antibiotic-resistant bacteria [58]. Thus, the fitness cost is associated with bacterial evolution and the maintenance of antibiotic resistance. The planktonic cell and biofilm cell numbers of ST^WT^ (*r* = 0.89) and ST^CI^ (*r* = 0.72) were highly correlated in the absence of PAβN. The numbers of active planktonic cells are associated with the surface-attached growth during biofilm maturation [59]. The adhesion of planktonic cells to an abiotic or biotic surface is critical at the early stage of biofilm formation [60]. The efflux pumps may be involved in biofilm formation such as the transport of extracellular polymeric substances (EPS) or QS signaling to facilitate biofilm matrix formation. Therefore, the inhibition of an efflux pump could also inhibit biofilm formation. No significant correlation was observed between ST^CI^ planktonic and biofilm cell numbers in the presence of PAβN (*r* = 0.23). This result suggests that the viability of planktonic cells was not influenced by PaβN, and the reduction in the biofilm cells was related to the inhibition of efflux pump developed in ST^CI^.

## 4. Materials and Methods

### 4.1. Bacterial Strains and Culture Conditions

Strains of *Salmonella enterica* subsp. *enterica* serovar Typhimurium ATCC 19585 (ST^WT^) and clinically isolated antibiotic-resistant *S*. Typhimurium CCARM 8009 (ST^CI^) were obtained from the American Type Culture Collection (ATCC; Manassas, VA, USA) and the Culture Collection of Antibiotic Resistant Microbes (CCARM; Seoul, Korea), respectively. *Chromobacterium violaceum* KACC 11542 was obtained from the Korean Agricultural Culture Collection (KACC; Seoul, Korea). Strains of ST^WT^ and ST^CI^ were cultivated in trypticase soy broth (TSB; Difco, BD, Sparks, MD, USA) at 37 °C for 20 h. C*. violaceum* was cultivated in Luria–Bertani (LB; Difco, BD, Sparks, MD, USA) broth at 26 °C for 20 h. The cultured cells were harvested by centrifugation at 6000× *g* for 10 min and diluted with phosphate-buffered saline (PBS, pH 7.2) to approximately 10^8^ CFU/mL for assays.

### 4.2. Antibiotic Susceptibility Assay

The susceptibilities of ST^WT^ and ST^CI^ to antibiotics and efflux pump inhibitor (EPI) were determined according to the Clinical Laboratory Standards Institute procedure with minor modifications [61]. Antibiotics (Table 1) including ceftriaxone (CEF), chloramphenicol (CHL), ciprofloxacin (CIP), erythromycin (ERY), norfloxacin (NOR), tetracycline (TET), and EPI (phenylalanine-arginine β-naphthylamide, PAβN) were purchased from Sigma Aldrich Chemicals (St. Louis, MO, USA). Antibiotic and EPI stock solutions were prepared by dissolving in sterile distilled water (CEF), ethanol (CHL, ERY, and TET), glacial acetic acid (CIP and NOR), and methanol (PAβN), respectively, at a concentration of 10.24 mg/mL. Each antibiotic stock solution (100 μL) was serially (1:2) diluted to concentrations ranging from 512 to 0.015 μg/mL in fresh TSB without and with PAβN at the pre-determined sub-minimum inhibitory concentrations (sub-MIC; 120 μg/mL) in 96-well microtiter plates (BD Falcon, San Jose, CA, USA). Each strain (10^5^ CFU/mL, 100 μL) was inoculated in the diluted antibiotic with EPI (1:2) and incubated at 37 °C for 18 h to determine MIC defined as the lowest concentrations of antibiotics where no visible bacterial growths were observed.

### 4.3. Biofilm-Forming Ability Assay

The biofilm-forming abilities of ST^WT^ and ST^CI^ (10^6^ CFU/mL each) treated with CEF, CHL, CIP, ERY, NOR, and TET were evaluated in the absence and presence of PAβN (120 μg/mL). The concentrations were determined at the 1/2 MICs of all antibiotics in the presence of PAβN, including CEF (0.0625 and 0.125 μg/mL), CHL (1 and 1 μg/mL), CIP (0.0078 and 0.0156 μg/mL), ERY (1 and 1 μg/mL), NOR (0.5 and 2 μg/mL), and TET (2 and 32 μg/mL) against ST^WT^ and ST^CI^, respectively. The biofilm cells were enumerated using a swabbing method with slight modification [62,63]. The test strains were also cultured in *C*. *violaceum*-cultured cell-free supernatant (CFS) in the absence and presence of PAβN (120 μg/mL). In brief, after 24 h of incubation at 37 °C, the biofilm cells were collected from the 96-well microtiter plates. Each well of the 96-well microtiter plate was gently rinsed two times with PBS to remove planktonic cells and scraped using a water-moistened sterile cotton swab. The collected biofilm cells harvested by centrifugation at 5000× *g* for 10 min at 4 °C were serially (1:10) diluted with PBS, plated on the TSA using an Autoplate^®^ Spiral Plating System (Spiral Biotech Inc., Norwood, MA, USA) and incubated at 37 °C for 24 h. The biofilm cell numbers were enumerated using a QCount^®^ Colony Counter (Spiral Biotech Inc.).

### 4.4. Growth-Based Bacterial Viability Assay

The antimicrobial activities of single antibiotic and combination treatments of antibiotics and PAβN against ST^WT^ and ST^CI^ were evaluated in TSB at 37 °C for 24 h. The initial population (10^7^ CFU/mL) of ST^WT^ and ST^CI^ was inoculated at 37 °C in *C*. *violaceum*-cultured CFS with 1/2 MICs of antibiotics in the absence and presence of PAβN (120 μg/mL). The antibiotics concentrations were 0.125 μg/mL CEF, 4 μg/mL CHL, 0.0156 μg/mL CIP, 128 μg/mL ERY, 2 μg/mL NOR, and 4 μg/mL TET for ST^WT^ in absence of PAβN, 0.0625 μg/mL CEF, 1 μg/mL CHL, 0.0078 μg/mL CIP, 1 μg/mL ERY, 0.5 μg/mL NOR, and 2 μg/mL TET for ST^WT^ in presence of PAβN, 0.125 μg/mL CEF, 4 μg/mL CHL, 0.03125 μg/mL CIP, 128 μg/mL ERY, 4 μg/mL NOR, and 256 μg/mL TET for ST^CI^ in the absence of PAβN, and 0.125 μg/mL CEF, 1 μg/mL CHL, 0.0156 μg/mL CIP, 1 μg/mL ERY, 2 μg/mL NOR, and 32 μg/mL TET for ST^CI^ in the presence of PAβN. The inoculated samples were incubated for 0, 4, 8, 12, and 24 h at 37 °C. After incubation, the collected planktonic cells were serially (1:10) diluted with PBS. Proper dilutions were plated on the TSA and incubated at 37 °C for 24 h. Viable cell numbers were enumerated as described above.

### 4.5. Biofilm-Forming Ability in CFS

ST^WT^ and ST^CI^ (10^7^ CFU/mL each) were cultured in *C. violaceum*-cultured CFS with 1/2 MICs of CEF, CHL, CIP, ERY, NOR, and TET with and without PAβN (120 μg/mL) in a 96-well plate. After incubation at 37 °C for 24 h, the cultures were centrifuged at 5000× *g* for 10 min at 4 °C to collect planktonic cells. Each well was rinsed with PBS, and biofilm cells were collected by using a cotton swab and centrifuged at 5000× *g* for 10 min at 4 °C. The planktonic and biofilm cells were enumerated as described above. The biofilm-forming index (BFI) was expressed as the ratio of number of planktonic cells to that of biofilm cells [64].

### 4.6. Swimming Motility Assay

Flagellum-dependent movement was determined by the swimming motility assay [65]. ST^WT^ and ST^CI^ cells were treated with 1/2 MICs of CEF, CHL, CIP, ERY, NOR, and TET in the presence and absence of PAβN (120 μg/mL) and incubated at 37 °C for 24 h. The planktonic cells (5 mL; 10^3^ CFU/mL) were collected and seeded onto the center of the surface of 0.3% agar-containing plates. After 12 h of incubation at 37 °C, the bacterial migration on each plate was measured using a digital vernier caliper (The L.S. Starrett Co., Athol, MA, USA).

### 4.7. Measurement of Relative Fitness

The relative fitness was estimated to assess the cost of resistance of ST^WT^ and ST^CI^ cells treated with 1/2 MICs of CEF, CHL, CIP, ERY, NOR, and TET in the presence and absence of PAβN (120 μg/mL) for 24 h. Then, the antibiotic-treated planktonic cells were collected by centrifugation at 5000× *g* for 10 min at 4 °C, rinsed two times with PBS, and cultured at 37 °C for 24 h in fresh TSB in the absence of antibiotics and PAβN. The relative fitness was expressed as the ratio of the growth (OD_600_) of treated cells to that of untreated control in the absence of antibiotics.

### 4.8. Statistical Analysis

All experiments were carried out in duplicate on three replicates. Data were analyzed using the Statistical Analysis System (SAS). The general linear model (GLM) and Fisher’s least significant difference (LSD) procedures were used to determine significant differences between treatments at *p* < 0.05, *p* < 0.01, and *p* < 0.001. The correlations between planktonic and biofilm cells were evaluated using Pearson’s correlation coefficient.

## 5. Conclusions

This study highlights the effect of efflux pump activity on the biofilm formation of *S*. Typhimurium. The most significant findings were that (1) the MICs of CEF, CHL, CIP, ERY, NOR, and TET against ST^WT^ and ST^CI^ were decreased in the presence of PAβN, (2) the sub-MICs of CEF, CHL, CIP, ERY, NOR, and TET were not effective in the inhibition of biofilm formation of ST^WT^ and ST^CI^ and, (3) PAβN significantly increased the inhibitory activity of sub-MICs of CEF, CHL, CIP, ERY, NOR, and TET on the biofilm formation by ST^WT^ and ST^CI^. The NOR+PAβN and CEF+PAβN treatments showed the highest anti-biofilm activity against ST^WT^ and ST^CI^, respectively. The inhibition of efflux pump by EPIs can interfere with the transport of quorum-sensing molecules, leading to the decrease in biofilm-forming abilities of pathogens. PaβN have no effect on the viability of planktonic cells but only inhibited biofilm formation. The use of antibiotics combined with EPIs has a great potential of controlling biofilm formation. Accordingly, EPIs can be used as a potential control agent to inhibit biofilm formation by pathogens.

## Figures and Tables

**Table 1:** Minimum inhibitory concentrations (MICs; µg/mL) of *Salmonella* Typhimurium ATCC 19585 (ST^WT^) and clinically isolated antibiotic-resistant *S.* Typhimurium CCARM 8009 (ST^CI^) in the absence and presence of phenylalanine-arginine-b-naphthylamide (PAβN).

Antibiotic	Inhibitory Mechanism	ST^WT^		ST^CI^	
		w/o PAβN	w/ PAβN	w/o PAβN	w/ PAβN
Ceftriaxone (CEF)	Cell wall synthesis	0.25	0.125	0.25	0.25
Chloramphenicol (CHL)	Protein synthesis	8	2	8	2
Ciprofloxacin (CIP)	DNA gyrase	0.031	0.016	0.063	0.031
Erythromycin (ERY)	Protein synthesis	256	2	256	2
Norfloxacin (NOR)	DNA gyrase	4	1	8	4
Tetracycline (TET)	Protein synthesis	8	4	512	64

## Data Availability

Not applicable.
